# Characterization of intercellular communication and mitochondrial donation by mesenchymal stromal cells derived from the human lung

**DOI:** 10.1186/s13287-016-0354-8

**Published:** 2016-07-12

**Authors:** Kenneth Andrew Sinclair, Stephanie Terase Yerkovich, Peter Mark-Anthony Hopkins, Daniel Charles Chambers

**Affiliations:** School of Medicine, University of Queensland, Brisbane, QLD Australia; Queensland Lung Transplant Service, Ground Floor, Clinical Sciences Building, The Prince Charles Hospital, Rode Road, Chermside, Brisbane, QLD 4032 Australia

**Keywords:** Mesenchymal stromal cells, Mesenchymal stem cells, Mitochondrial donation, Intercellular communication, Bronchoalveolar lavage

## Abstract

**Background:**

Bone marrow-derived mesenchymal stromal cells (BM-MSCs) are capable of repairing wounded lung epithelial cells by donating cytoplasmic material and mitochondria. Recently, we characterized two populations of human lung-derived mesenchymal stromal cells isolated from digested parenchymal lung tissue (LT-MSCs) from healthy individuals or from lung transplant recipients’ bronchoalveolar lavage fluid (BAL-MSCs). The aim of this study was to determine whether LT-MSCs and BAL-MSCs are also capable of donating cytoplasmic content and mitochondria to lung epithelial cells.

**Methods:**

Cytoplasmic and mitochondrial transfer was assessed by co-culturing BEAS2B epithelial cells with Calcein AM or Mitotracker Green FM-labelled MSCs. Transfer was then measured by flow cytometry and validated by fluorescent microscopy. Molecular inhibitors were used to determine the contribution of microtubules/tunnelling nanotubes (TNTs, cytochalasin D), gap junctions (carbenoxolone), connexin-43 (gap26) and microvesicles (dynasore).

**Results:**

F-actin microtubules/TNTs extending from BM-MSCs, LT-MSCs and BAL-MSCs to bronchial epithelial cells formed within 45 minutes of co-culturing cells. Each MSC population transferred a similar volume of cytoplasmic content to epithelial cells. Inhibiting microtubule/TNTs, gap junction formation and microvesicle endocytosis abrogated the transfer of cytoplasmic material from BM-MSCs, LT-MSCs and BAL-MSCs to epithelial cells. In contrast, blocking connexin-43 gap junction formation had no effect on cytoplasmic transfer. All MSC populations donated mitochondria to bronchial epithelial cells with similar efficiency. Mitochondrial transfer was reduced in all co-cultures after microtubule/TNT or endocytosis inhibition. Gap junction formation inhibition reduced mitochondrial transfer in BM-MSC and BAL-MSC co-cultures but had no effect on transfer in LT-MSC co-cultures. Connexin-43 inhibition did not impact mitochondrial transfer. Finally, bronchial epithelial cells were incapable of donating cytoplasmic content or mitochondria to any MSC population.

**Conclusion:**

Similar to their bone marrow counterparts, LT-MSCs and BAL-MSCs can donate cytoplasmic content and mitochondria to bronchial epithelial cells via multiple mechanisms. Given that BM-MSCs utilize these mechanisms to mediate the repair of damaged bronchial epithelial cells, both LT-MSCs and BAL-MSCs will probably function similarly.

**Electronic supplementary material:**

The online version of this article (doi:10.1186/s13287-016-0354-8) contains supplementary material, which is available to authorized users.

## Background

Recent studies suggest that adult stem cells reside within niches where they coordinate organ homeostasis and repair after injury. In the bone marrow, mesenchymal stromal cells (MSCs) and haematopoietic stem cells (HSCs) reside proximal to adrenergic nerve fibres to form the HSC niche [1]. Bone marrow-derived MSCs (BM-MSCs) are an essential component of this structure, forming gap junctions with HSCs and expressing HSC maintenance genes which directly contribute to the HSC volume and capacity to mobilize to extramedullary sites during haematopoiesis [1]. Emerging evidence suggests that the function of MSCs within stem cell niches, irrespective of organ, is to provide support for and coordinate the behaviour of parenchymal progenitor cells [1–4]. In order to accomplish this, BM-MSCs must be able to effectively communicate and interact with their target cells. Known mechanisms already characterized for BM-MSCs include microtubules [5], tunnelling nanotubes (TNTs) [3, 6–8], gap junctions [9], microvesicles [9–12] and exosomes [13, 14]. While the transferred material is yet to be fully defined, it is known to include growth factors [1, 7], microRNA [14], immuno-modulatory molecules [15–17] and mitochondria [5, 6, 8, 9]. Notably, mitochondrial transfer is emerging as an important mechanism by which MSCs can stabilize or enhance the bioenergetic potential of target cells, which includes alveolar epithelial cells [9] and alveolar macrophages [18]. These mechanisms are thought to contribute to the therapeutic benefit of MSCs by enhancing epithelial proliferation [5, 9], modulating T-lymphocyte proliferation [19, 20] and inducing the polarization of macrophages towards the M2 phenotype [18, 21, 22]. As such, MSCs have been demonstrated to promote tissue homeostasis and tissue repair in multiple models of lung injury including heat shock [5, 23], cigarette smoke-mediated wounding [6], acute lung injury [8, 9], experimental asthma [8, 24, 25] and bleomycin-induced pulmonary fibrosis [26, 27]. Despite this knowledge, whether these modes of behaviour exist in MSC populations which reside endogenously within the human lung remains to be elucidated.

The relationship between MSCs and parenchymal progenitor cells is yet to be fully described in the human lung, but is probably analogous to that of the bone marrow. We suspected that human lung MSCs promote tissue homeostasis and repair by donating cytoplasmic material and mitochondria to parenchymal cells such as bronchial epithelium. Evidence to support this hypothesis comes from murine studies which demonstrate that lung-derived MSCs produce growth factors such as fibroblast growth factor-10 and hepatocyte growth factor, which promote the proliferation and propagation of lung epithelial progenitor cells into complex airway and alveolar structures [2, 28]. Recently, we described two populations of human lung MSCs isolated from digested parenchymal lung tissue (LT-MSCs) from healthy individuals or from lung transplant recipients’ bronchoalveolar lavage fluid (BAL-MSCs) [30]. The aim of this study was to test the hypothesis that, like their bone marrow counterparts, human lung-derived MSCs donate cytoplasmic content and mitochondria to human bronchial epithelial cells, in vitro, and to characterize the underlying mechanisms.

## Method

### Cell lines and ethics

Human BM-MSCs were kindly donated by Cell and Tissue Therapies, WA, Australia. BEAS2B epithelial cell lines were purchased from American Type Tissue Culture Collection.

### LT-MSC isolation

Parenchymal lung tissue was obtained from patients undergoing a blebectomy to treat recurrent pneumothorax. Samples of macroscopically normal lung were mechanically digested into 1 mm^3^ pieces and seeded onto plastic culture dishes at a density of 1 g per dish (10 cm diameter). Cells were maintained in Dulbecco’s modified Eagle medium (DMEM; Gibco), supplemented with 10 % fetal calf serum and penicillin/streptomycin/glutamine (Gibco). Between 7 and 14 days after seeding, colony-forming units comprised of LT-MSCs were observable. MSC colonies were isolated and transferred to individual flasks where they were allowed to clonally expand up to 60–80 % confluence, after which they were cryopreserved in liquid nitrogen. All cells used were between passages 2 and 4 and were preserved in liquid nitrogen prior to use.

### BAL-MSC isolation

We and others have reported previously that BAL-MSCs are rarely isolated from healthy individuals [29, 30]. Bronchoalveolar lavage (BAL) fluid was therefore collected from lung transplant recipients undergoing a post-transplant bronchoscopy. BAL was performed by wedging the bronchoscope in the middle lobe or lingula, infusing 100 ml of 0.9 % saline via the working channel and aspirating the effluent. BAL samples were immediately centrifuged and seeded into six-well plates at a density of (0.3-0.4) × 10^6^ cells/well. Colony-forming units comprised of BAL-MSCs were observable within 14–21 days after seeding. Cells were expanded and maintained as already detailed.

### MSC characterization

As described previously [29, 31], BAL-MSCs and LT-MSCs are classified as MSCs based on the diagnostic criteria outlined by The Internationally Society for Cellular Therapy [32] and transcriptional profiling [33]. Briefly, both populations are plastic adherent, express the cell surface proteins CD105, CD73 and CD90, and lack CD45 expression. Cells also have a demonstrable capacity for osteoblast, chondroblast and adipocyte differentiation [29, 30, 31]. Finally, both populations qualify as MSCs according to transcriptional profiling [30, 33]. Additionally, BAL-MSCs lack characteristic fibrocyte markers and express a HLA serotype consistent with that of the lung donor.

### Cytoplasmic exchange and mitochondrial donation assays

BEAS2B lung epithelial cells were labelled with 5 μl of DiD labelling solution (Life Technologies) for 20 minutes at 37 °C. MSCs were labelled with 1 mM Calcein AM (Life Technologies) for 35 minutes at 37 °C or 40 nM Mitotracker Green (Life Technologies) for 20 minutes at 37 °C. Cells were cultured in 24-well sized plates pre-coated with a solution of 0.1 mg/ml fibronectin (Sigma Aldrich), 0.03 mg/ml bovine collagen type 1 (Worthington) and 0.01 mg/ml bovine serum albumin (Bovogen). Then 3 × 10^4^ BEAS2B cells were seeded and allowed to adhere overnight, prior to the addition of 3 × 10^4^ MSCs. Cells were co-cultured for 4 hours in a 1:1 mixture of basal epithelial growth medium (BEGM; Lonza) and DMEM containing 10 % fetal calf serum. Cells were then washed with phosphate-buffered saline (PBS) and detached by incubating cells in 0.25 % trypsin (Gibco) for 5 minutes at 37 °C. Prior to flow cytometric assessment, cellular viability was assessed using 4 % trypan blue exclusion and light microscopy. Cellular viability was always greater than 95 %. For transwell assays, all details are as already listed except MSCs were seeded into 0.4 μM transwell inserts (MilliCell). Specific dye transfer was measured by gating on DiD^Pos^ events, excluding doublets (forward scatter width vs height) and measuring fluorescence in the fluorescein channel (Additional file 1: Figure S1 and Additional file 2: Figure S2). The mean fluorescent intensity (MFI) is expressed as a ratio to unstained controls. Flow cytometry was performed on a FACS CantoA (BD) and data were analysed using FloJo (Tree Star). Each data point shown in Figs. 2, 3, 4 and 5 represents the average of two replicates.

**Fig. 1:**
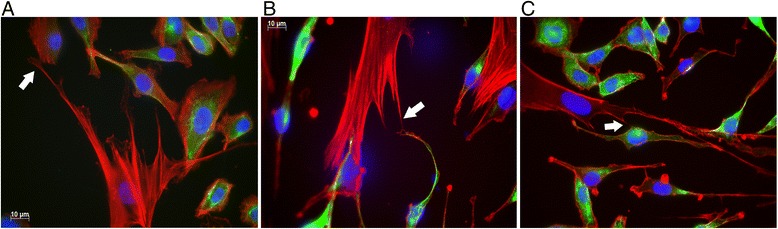
BM-MSCs, LT-MSCs and BAL-MSCs connect with BEAS2B bronchial epithelial cells through F-actin containing microtubules. Representative immunofluorescent images of (**a**) BM-MSCs, (**b**) LT-MSCs and (**c**) BAL-MSCs co-cultured with BEAS2B epithelial cells. Epithelial cells (*green*) were labelled with cytokeratin and all cells were counterstained with phalloidin (F-actin, *red*). Cell nuclei were labelled with DAPI (*blue*). Images shown at 630× magnification. *White arrows* depict microtubules connecting MSCs and epithelial cells

**Fig. 2:**
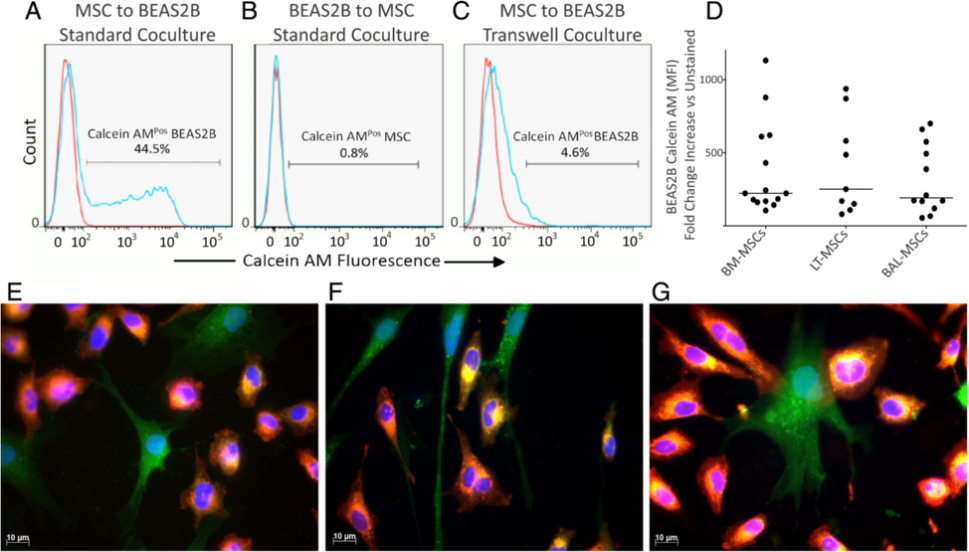
BM-MSCs, LT-MSCs and BAL-MSCs donate cytoplasmic contents to BEAS2B epithelial cells. Cytoplasmic transfer between mesenchymal stromal cells (*MSCs*) and BEAS2B bronchial epithelial cells was measured by flow cytometry and immunofluorescence. **a** Representative flow cytometry plot of transfer after MSCs were labelled with Calcein AM and co-cultured with BEAS2B cells for 4 hours. *Blue line* represents co-cultured BEAS2B cells, *red line* represents unstained control. **b** To assess whether epithelial cells were capable of donating cytoplasmic material to MSCs, BEAS2B cells were labelled with Calcein AM and co-cultured with MSCs for 4 h. *Blue line* represents co-cultured MSCs, *red line* represents unstained control. **c** Representative flow cytometry plot of transfer after MSCs were labelled with Calcein AM and co-cultured with BEAS2B cells within a transwell configuration.  **d** Grouped data of cytoplasmic transfer by bone marrow-derived (*BM-MSCs*), lung tissue-derived (*LT-MSCs*) and bronchoalveolar fluid-derived (*BAL-MSCs*) MSCs. *Y* axis represents the fold-change increase in Calcein AM mean fluorescent intensity (*MFI*) of BEAS2B cells after co-culture with each respective MSC population. *Horizontal bars* represent median. **e**–**g** Representative immunofluorescent images of cytoplasmic transfer by BM-MSCs (**e**), LT-MSCs (**f**) and BAL-MSCs (**g**). MSCs were pre-labelled with Calcein AM (*green*) and co-cultured with BEAS2B epithelial cells labelled with DiD (*red*). BEAS2B cells containing MSC-derived Calcein AM appear *yellow/orange*. Images shown at 630× magnification. Scale bars represent 10 μM

**Fig. 3:**
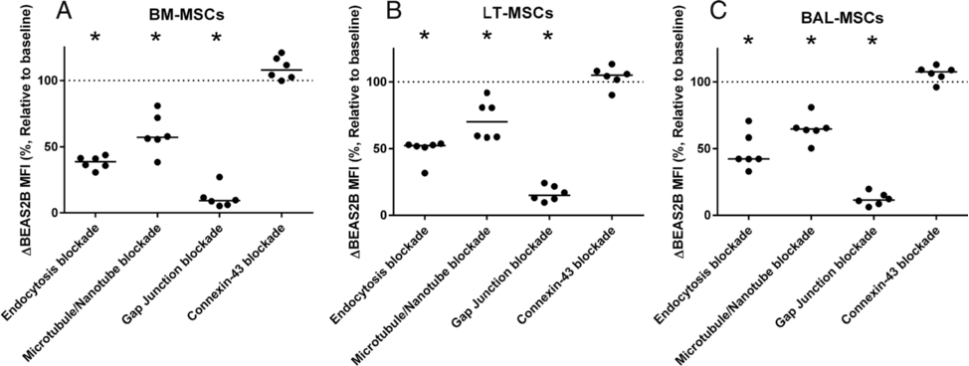
BM-MSCs, LT-MSCs and BAL-MSCs donate cytoplasmic contents to BEAS2B epithelial cells via multiple mechanisms. Bone marrow-derived mesenchymal stromal cells (*BM-MSCs*, **a**), lung tissue-derived mesenchymal stromal cells (*LT-MSCs*, **b**) and bronchoalveolar fluid-derived mesenchymal stromal cells (*BAL-MSCs*, **c**) were co-cultured with BEAS2B epithelial cells and treated with either dynasore to inhibit endocytosis, cytochalasin D to inhibit microtubule/TNT formation, carbenoxolone to inhibit all gap junction formation or GAP26 to inhibit connexin-43 gap junction formation. *Y* axis represents this change in mean fluorescent intensity (*MFI*, %) relative to paired, uninhibited control. *Horizontal line* represents median. Groups were compared using the Mann–Whitney *U* test. *p < 0.05 compared with baseline

**Fig. 4:**
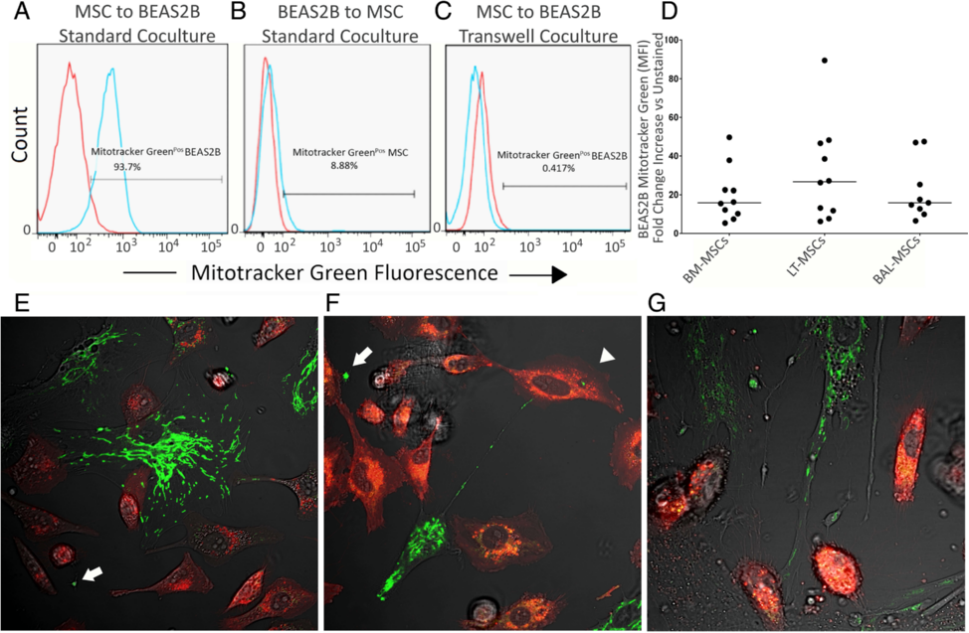
BM-MSCs, LT-MSCs and BAL-MSCs donate mitochondria to BEAS2B epithelial cells. Mitochondrial transfer between mesenchymal stromal cells (*MSCs*) and BEAS2B bronchial epithelial cells was measured by flow cytometry and confocal microscopy. **a** Representative flow cytometry plot of transfer after MSCs were labelled with Mitotracker Green and co-cultured with BEAS2B cells for 4 hours. *Blue line* represents co-cultured BEAS2B cells, *red line* represents unstained control. **b** To assess whether epithelial cells were capable of donating mitochondrial to MSCs, BEAS2B cells were labelled with Mitotracker Green and co-cultured with MSCs for 4 h. *Blue line* represents co-cultured MSCs, *red line* represents unstained control. **c** Representative flow cytometry plot of transfer after MSCs were labelled with Mitotracker Green and co-cultured with BEAS2B cells within a transwell configuration. **d** Grouped data of mitochondrial transfer by bone marrow-derived (*BM-MSCs*), lung tissue-derived (*LT-MSCs*) and bronchoalveolar fluid-derived (*BAL-MSCs*) MSCs. *Y* axis represents the fold-change increase in Mitotracker Green mean fluorescent intensity (*MFI*) of BEAS2B cells after co-culture with each respective MSC population. *Horizontal bars* represent median. **e**–**g** Confocal microscopy images of mitochondrial transfer by BM-MSCs (**e**), LT-MSCs (**f**) and BAL-MSCs (**g**). MSCs were pre-labelled with Mitotracker Green (*green*) and co-cultured with BEAS2B epithelial cells labelled with DiD (*red*). Images are shown at 630× magnification with an oil immersion lens. *White arrows* point to microvesicles containing mitochondria, *white arrowhead* points to an endocytosed MSC-derived mitochondria

**Fig. 5:**
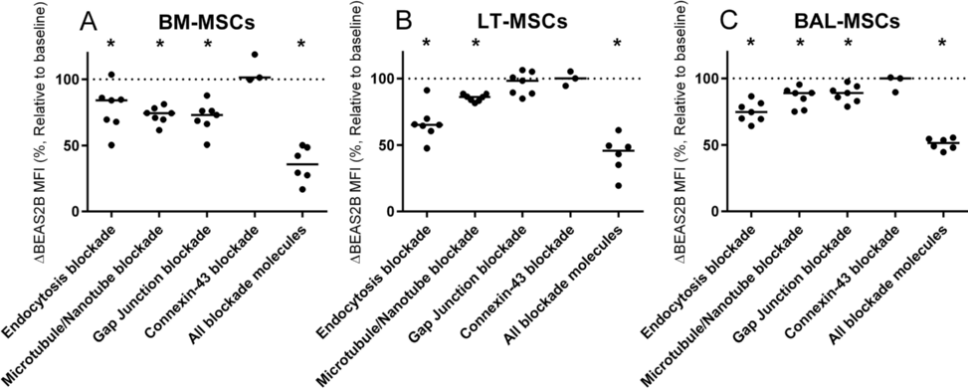
BM-MSCs, LT-MSCs and BAL-MSCs donate mitochondria to BEAS2B epithelial cells. Bone marrow-derived mesenchymal stromal cells (*BM-MSCs*, **a**), lung tissue-derived mesenchymal stromal cells (*LT-MSCs*, **b**) and bronchoalveolar fluid-derived mesenchymal stromal cells (*BAL-MSCs*, **c**) were cultured with either dynasore to inhibit endocytosis, cytochalasin D to inhibit microtubule/nanotube formation, carbenoxolone to inhibit all gap junction formation or GAP26 to inhibit connexin-43 gap junction formation. *Y* axis represents this change in mean fluorescent intensity (*MFI*, %) relative to a paired, uninhibited control. *Horizontal line* represents median. Groups were compared using the Mann–Whitney *U* test. *p < 0.05 compared with baseline

Inhibitory compounds (or vehicle controls; Additional file 3: Table S1) used to determine the mechanisms underlying cytoplasmic/mitochondrial transfer were added to culture simultaneously with MSCs and remained in culture for the duration of the assay.

### Fluorescent microscopy

Co-cultures were fixed with 4 % paraformaldehyde (BD), permeabilized with 1 % Triton X (Scharlau) in PBS and blocked for 1 hour in 1 % bovine serum albumin (Bovogen). Co-cultured cells were stained with Phalloidin (AbCam CytoPainter Kit) for 90 minutes for microtubules/TNTs, as per the manufacturer’s instructions, and α-cytokeratin-FITC (15 μG; Milltenyi) for epithelial cells, washed with PBS and mounted in ProLong Gold (containing DAPI; Life Technologies). Fluorescent labelling was visualized using an Axiolmager Z1 microscope (Zeiss) and Axiovision image acquisition software. For live cell confocal microscopy, MSCs were labelled with Mitotracker Green FM and BEAS2B cells with DiD, as already described. Cells were seeded onto 10 mm diameter glass-bottom culture dishes (Ibidi). Dishes were imaged at 37 °C at a rate of 1 image per 10 seconds using a 63× oil immersion lens, coupled with a 10× eyepiece lens and a differential interference contrast filter using a Leica TCS SP2 microscope.

### Statistics

Statistical analysis was performed using Stata v11 (StataCorp) with the Mann–Whitney *U* test or Wilcoxon signed-rank test, as appropriate. All data are expressed as median and interquartile range unless otherwise stated. Statistical significance was defined as *p* < 0.05.

## Results

### Patient demographics

Healthy parenchymal lung tissue was collected from 10 patients (six male) with a median age of 24.9 (21.7–27.9) years. BAL-MSCs were collected from 14 lung transplant recipients (10 male) with a median donor age of 30 (28–54) years and median time post transplant of 2.78 (0.27–4.48) years. Patient demographics for BAL-MSCs are presented in Table 1.

**Table 1:** Patient demographics for bronchoalveolar lavage-derived mesenchymal stromal cells

	Lung transplant patients (*n* = 14)
Age at transplantation (years)	38.75 (30.0–56.0)
Male sex	10 (71.4)
Donor age (years)	30 (28–54)
Male donor sex	10 (71.4)
Time post transplant (years)	2.78 (0.27–4.48)
Pre-transplant diagnosis	
Cystic fibrosis	6 (42.8)
Chronic obstructive pulmonary disease	4 (28.5)
Idiopathic pulmonary fibrosis	2 (14.2)
Other	2 (14.2)
Transplant type	
Single	1 (7.1)
Double	12 (85.7)
Heart/lung	1 (7.1)

Data presented as median (interquartile range) or *n* (%)

### BM-MSCs, LT-MSCs and BAL-MSCs connect with BEAS2B epithelial cells via microtubules and TNTs

Microtubules and TNTs were first observed connecting MSCs and BEAS2B epithelial cells after 45 minutes of co-culturing cells. These projections stained strongly with phalloidin indicating that they are comprised of F-actin, consistent with previous reports for BM-MSCs [8] (Fig. 1).

### BM-MSCs, LT-MSCs and BAL-MSCs have a similar efficiency for uni-directional cytoplasmic transfer to BEAS2B epithelial cells

We aimed to determine whether the observed interaction between MSCs and epithelial populations mediated the transfer of cytoplasmic material. Calcein AM transfer from MSCs to epithelial cells was rapid and first observed after 4 hours of co-culture, as shown by an increase in BEAS2B cell Calcein AM fluorescence (Fig. 2) and a corresponding decrease in MSC Calcein AM fluorescence (Additional file 4: Figure S3A).

Cytoplasmic transfer occurred at a similar level for all MSC populations (Fig. 2d). After co-culture with BM-MSCs, the Calcein AM MFI in BEAS2B cells was increased 223.5-fold (172.2–565.9, *n* = 14). Similarly, BEAS2B Calcein AM MFI was increased 252.1-fold (150.9–252.1, *n* = 9) after co-culture with LT-MSCs and was increased 192.3-fold (156.9–513.4, *n* = 12) after co-culture with BAL-MSCs. We did not observe Calcein AM transfer when the cells were separated by transwell culture (Fig. 2c). Finally, cytoplasmic transfer was uni-directional and could not be observed to occur from BEAS2B cells to any MSC population (Fig. 2b).

### Cytoplasmic transfer from BM-MSCs, LT-MSCs and BAL-MSCs to BEAS2B epithelial cells is mediated by contact-dependent and contact-independent mechanisms

We next sought to characterize the mechanisms of cytoplasmic transfer between MSCs and epithelial cells (Fig. 3). In line with a previous report [9], we used the dynamin inhibitor, Dynasore, to inhibit BEAS2B cells endocytosing MSC-produced microvesicles. Endocytosis inhibition reduced cytoplasmic transfer by 61.3 % (58.7–64.1), 47.7 % (47.1–48.7) and 57.7 % (45.68–57.7) in BM-MSC, LT-MSC and BAL-MSC co-cultures, respectively (*n* = 6, *p* < 0.05 for all; Fig. 3). Blocking microtubule/TNT formation using the actin polymerase inhibitor cytochalasin D [34] reduced cytoplasmic transfer by 43.0 % (31.7–44.3), 40.5 % (19.3–41.2) and 36.0 % (34.6–39.4) from BM-MSCs, LT-MSCs and BAL-MSCs, respectively (*n* = 6, *p* < 0.05 for all). The greatest reduction in cytoplasmic transfer was observed using the non-specific gap junction blocker carbenoxolone [35, 36] (Fig. 3). Cytoplasmic transfer from BM-MSCs to BEAS2B cells was reduced by 90.8 % (88.9–93.2, *p* < 0.05, *n* = 6) after carbenoxolone treatment. Similarly, transfer was reduced by 85.0 % (79.4–87.5, *p* < 0.05, *n* = 6) and 88.5 % (85.6–90.9, *p* < 0.05, *n* = 6) in LT-MSC and BAL-MSC co-cultures respectively. To determine the contribution of connexin-43-dependent gap junctions, which have previously been demonstrated to mediate cytoplasmic transfer in murine studies [9], we employed the inhibitor gap26 [37, 38]. Blocking connexin-43 did not prevent cytoplasmic transfer with any MSC cell type.

### BM-MSCs, LT-MSCs and BAL-MSCs share similar efficiency for uni-directional mitochondrial transfer to BEAS2B epithelial cells

BM-MSCs can promote the regeneration of damaged lung epithelial cells through the donation of mitochondria using similar mechanisms to those described for cytoplasmic transfer [5, 9]. We found that all MSC populations can donate mitochondria to BEAS2B epithelial cells, as indicated by increased BEAS2B Mitotracker Green fluorescence (Fig. 4) and decreased MSC Mitotracker Green fluorescence (Additional file 4: Figure S3B) after co-culture. When visualized using live cell confocal microscopy, mitochondria localized within MSC microtubules or TNTs, tracked towards BEAS2B cells and crossed the cell membrane border. In addition, individual mitochondrion could be seen within endosomes (Fig. 4e, white arrowhead) and microvesicles (Fig. 4e, f, white arrows). Following co-culture with BM-MSCs, BEAS2B Mitotracker Green MFI increased 15.9-fold (10.7–22.4, *n* = 10). Similarly, BEAS2B Mitotracker Green MFI increased 26.7-fold (12.1–44.6, *n* = 10) after co-culture with LT-MSCs and increased 15.9-fold (12.7–25.2, *n* = 9) after co-culture with BAL-MSCs (Fig. 4d). Mitochondrial transfer was not observed when BEAS2B cells were co-cultured with any MSC populations using a transwell set-up (Fig. 4c). Additionally, we did not observe any mitochondrial transfer from BEAS2B cells to any MSC populations (Fig. 4b).

### Mitochondrial transfer from BM-MSCs, LT-MSCs and BAL-MSCs to BEAS2B epithelial cells is mediated by contact-dependent and non-dependent mechanisms

Inhibiting endocytosis in BEAS2B cells resulted in a 15.8 % (14.6–31.2, *p* < 0.05, *n* = 7) decrease in transfer in BM-MSC co-cultures (Fig. 5). Mitochondrial donation was also decreased after endocytosis inhibition by 34.7 % (32.3–37.4, *p* < 0.05, *n* = 7) in LT-MSCs and by 25.2 % (20.0–30.3, *p* < 0.05, *n* = 7) in BAL-MSC co-cultures (Fig. 5). Inhibiting microtubule/TNT formation reduced mitochondrial transfer by 25.5 % (23.6–29.7, *p* < 0.05, *n* = 7) in BM-MSC co-cultures. Transfer was reduced by 13.8 % (12.4–16.2, *p* < 0.05, *n* = 7) in LT-MSC co-cultures and by 11.0 % (10.0–30.3, *p* < 0.05, *n* = 7) in BAL-MSC co-cultures. The non-specific inhibition of gap junction formation reduced mitochondrial donation by 26.8 % (23.8–32.5, *p* < 0.05, *n* = 7) in BM-MSC co-cultures and by 10.9 % (7.5–15.6, *p* < 0.05, *n* = 7) in BAL-MSC co-cultures. Gap junction inhibition did not reduce mitochondrial donation in LT-MSC co-cultures. Finally, connexin-43 inhibition did not reduce mitochondrial transfer in any MSC-BEAS2B cell co-culture (Fig. 5). When all inhibitory compounds were added together we observed greater attenuation of mitochondrial transfer (Fig. 5), suggesting synergy between microtubule/TNT, endocytosis and gap junction mediated mechanisms.

## Discussion

MSCs exhibit therapeutic effects in multiple models of acute and chronic lung epithelial injury and represent a promising therapeutic approach for the treatment of end-stage lung diseases including idiopathic pulmonary fibrosis [39] and acute lung injury [40] in humans. These studies have utilized MSCs isolated from tissue sources such as the bone marrow, adipose and placenta. Comparative studies have demonstrated that, irrespective of source, MSCs from any two tissues are strikingly similar [41, 42]. Despite this, each population possesses subtle nuances, specific to their source [30, 43], which may confer additional benefit or efficiency in a therapeutic context. One of the mechanisms by which MSCs support epithelial cell repair is thought to be via direct transfer of cytoplasmic contents [9, 35], but the nature of the transferred material, mechanisms of transfer, effects on target cells and effects of the tissue microenvironment have yet to be fully defined. In this study, we demonstrate that human MSCs isolated from bone marrow, parenchymal lung tissue and BAL fluid donate cytoplasmic contents and mitochondria to bronchial epithelial cells. Transfer was rapid, uni-directional and mediated by multiple mechanisms including microvesicles, microtubules/TNTs and non-connexin-43 gap junctions. Similar mechanisms contributed to mitochondrial donation in BM-MSCs and BAL-MSCs, whereas microvesicles and microtubules/TNTs, but not gap junctions, contributed to mitochondrial transfer by LT-MSCs. Inhibiting these pathways did not completely prevent mitochondrial donation, however, suggesting the existence of additional mechanisms. These findings add to a growing body of literature which demonstrates MSCs isolated from different sources are similar but exhibit subtle differences which may be reflective of adaption to a specific tissue. Our data provide further support to the idea that human lung-derived mesenchymal populations contribute to tissue homeostasis and repair through direct interaction with epithelial cell populations.

BAL-MSCs are a unique population of transplant donor-derived cells that are generally only recoverable from lung allografts [29, 30]. Their absence from healthy individuals and correlation with disease has led to the suggestion that BAL-MSCs are either a causal factor for lung disease [44, 45] or an epiphenomenon. We have previously postulated that BAL-MSCs are derived from an in-tissue source which has mobilized into the alveolar space to provide support for injured epithelial cells [30]. This is supported by previous studies demonstrating that BAL-MSCs administered to mice preferentially engraft in close proximity to alveolar progenitor cells and donate keratinocyte growth factor via connexin-43 gap junctions [35]. Our findings expand on this by demonstrating that cytoplasmic content is transferred from human LT-MSCs and BAL-MSCs to human bronchial epithelial cells and by identifying a capacity for mitochondrial donation. Unlike previous studies, we were unable to identify a role of connexin-43 in the transfer of cytoplasmic content or mitochondria. These findings provide a foundation for future investigations into the reparative capacity of BAL-MSCs and LT-MSCs.

Although it is now clear that LT-MSCs and BAL-MSCs transfer cytoplasmic material to the lung epithelium, the composition of transferred material remains to be characterized. We hypothesize that LT-MSCs and BAL-MSCs transfer material analogous to that produced by BM-MSCs. While this material remains incompletely defined, it is known to include growth factors [26, 46], immuno-modulatory molecules [16, 47], transcription factors [12] and micro-RNAs [11, 14]. Human BAL-MSCs are thought to transfer keratinocyte growth factor to murine type 2 alveolar epithelial cells, in vivo [35]. Additionally, murine LT-MSCs have been demonstrated to produce fibroblast growth factor-10 and hepatocyte growth factor which drives the differentiation of epithelial progenitor cells into complex airway and alveolar structures, in vitro [2]. Together, these findings support the notion that the role of MSCs within tissue is to provide support, be it by the production of paracrine factors or mitochondrial donation to the surrounding parenchymal populations.

Our data suggest that human BM-MSCs and BAL-MSCs employ a non-connexin-43 gap junction in the transfer of cytoplasmic content and mitochondria to bronchial epithelial cells. This finding was surprising as connexin-43 has previously been demonstrated to mediate both cytoplasmic and mitochondrial transfer from BM-MSCs to alveolar epithelial cells [9]. Together, these findings suggest MSCs utilize gap junctions in a region-specific manner. To date, no other connexins have been implicated in the transfer of cytoplasmic content or mitochondria. The most logical alternative candidate is connexin-26 which is expressed by basal cells [48]. Notably, basal cells serve as a progenitor cell for the bronchial epithelium [49] and interaction between basal cells and local MSCs would fit with the developing narrative of the pulmonary stem cell niche.

There are several limitations to this study. Firstly, our study used a human bronchial epithelial cell line which may limit the generalisability of our findings to primary cells. This may explain why we were unable to demonstrate a role for connexin-43 in cytoplasmic or mitochondrial donation. However, healthy human lung epithelium is difficult to obtain and requires a heavily supplemented growth medium, which in itself can lead to artefacts. Secondly, the isolation and ex-vivo expansion of MSCs can lead to functional alterations, including their interaction with lung epithelial cells [2]. Therefore, we used MSCs at the lowest passage feasibly possible and passage-matched all samples.

## Conclusion

LT-MSCs and BAL-MSCs share a similar capacity for cytoplasmic and mitochondrial transfer to bronchial epithelial cells. Transfer is rapid, ubiquitous and unidirectional from MSCs to the epithelium. These findings offer novel insight into the potential role of lung MSCs in organ homeostasis and repair.

## Abbreviations

BAL, bronchoalveolar lavage; BAL-MSC, bronchoalveolar lavage-derived mesenchymal stromal cell; BEGM, basal epithelial growth medium; BM-MSC, bone marrow-derived mesenchymal stromal cell; DMEM, Dulbecco’s modified Eagle medium; HSC, haematopoietic stem cell; LT-MSC, lung tissue-derived mesenchymal stromal cell; MFI, mean fluorescent intensity; MSC, mesenchymal stromal cell; PBS, phosphate-buffered saline; TNT, tunnelling nanotube

## Additional files

Additional file 1: Figure S1. Showing the gating strategy for measuring BEAS2B Calcein AM fluorescence after co-culture. MFI of Calcein AM within BEAS2B cells was determined by flow cytometry. (a) Forward/side scatter gating was used to exclude debris. (b) BEAS2B epithelial cells were then selected based on DiD fluorescence. (c) Doublets were then eliminated using forward scatter—width and height. (d) A fluorescence minus one control (*red line*) was used to distinguish positive Calcein AM and negative BEAS2B cells. (TIF 481 kb) Additional file 2: Figure S2. Showing the gating strategy for measuring BEAS2B Mitotracker Green fluorescence. MFI of Mitotracker Green within BEAS2B cells was determined by flow cytometry. (a) Forward/side scatter gating was used to exclude debris. (b) BEAS2B epithelial cells were then selected based on DiD fluorescence. (c) Doublets were then eliminated using forward scatter—width and height. (d) A fluorescence minus one control (*red line*) was used to distinguish positive Mitotracker Green and negative BEAS2B cells. (TIF 461 kb) Additional file 3: Table S1. Presenting supplementary methods. (DOCX 12 kb) Additional file 4: Figure S3. Showing that MSC Mitotracker Green fluorescence decreases after co-culture with BEAS2B epithelial cells. The change in mesenchymal stromal cell fluorescence (a Calcein AM, b Mitotracker Green) after co-culture with BEAS2B epithelial cells. *Dotted horizontal line* represents baseline calculated as the MFI of MSC grown in homogenous cultures. *Solid horizontal line* represents median. (TIF 20 kb)

## References

[1] Mendez-Ferrer S, Michurina TV, Ferraro F, Mazloom AR, Macarthur BD, Lira SA (2010). Mesenchymal and haematopoietic stem cells form a unique bone marrow niche. Nature.

[2] McQualter JL, McCarty RC, Van der Velden J, O’Donoghue RJ, Asselin-Labat ML, Bozinovski S (2013). TGF-beta signaling in stromal cells acts upstream of FGF-10 to regulate epithelial stem cell growth in the adult lung. Stem Cell Res.

[3] Acquistapace A, Bru T, Lesault PF, Figeac F, Coudert AE, le Coz O (2011). Human mesenchymal stem cells reprogram adult cardiomyocytes toward a progenitor-like state through partial cell fusion and mitochondria transfer. Stem Cells.

[4] Munoz JR, Stoutenger BR, Robinson AP, Spees JL, Prockop DJ (2005). Human stem/progenitor cells from bone marrow promote neurogenesis of endogenous neural stem cells in the hippocampus of mice. Proc Natl Acad Sci U S A.

[5] Spees JL, Olson SD, Whitney MJ, Prockop DJ (2006). Mitochondrial transfer between cells can rescue aerobic respiration. Proc Natl Acad Sci U S A.

[6] Li X, Zhang Y, Yeung SC, Liang Y, Liang X, Ding Y (2014). Mitochondrial transfer of induced pluripotent stem cell-derived mesenchymal stem cells to airway epithelial cells attenuates cigarette smoke-induced damage. Am J Respir Cell Mol Biol.

[7] Figeac F, Lesault PF, Le Coz O, Damy T, Souktani R, Trebeau C (2014). Nanotubular crosstalk with distressed cardiomyocytes stimulates the paracrine repair function of mesenchymal stem cells. Stem Cells.

[8] Ahmad T, Mukherjee S, Pattnaik B, Kumar M, Singh S, Kumar M (2014). Miro1 regulates intercellular mitochondrial transport & enhances mesenchymal stem cell rescue efficacy. EMBO J.

[9] Islam MN, Das SR, Emin MT, Wei M, Sun L, Westphalen K (2012). Mitochondrial transfer from bone-marrow-derived stromal cells to pulmonary alveoli protects against acute lung injury. Nat Med.

[10] Zhu YG, Feng XM, Abbott J, Fang XH, Hao Q, Monsel A (2014). Human mesenchymal stem cell microvesicles for treatment of Escherichia coli endotoxin-induced acute lung injury in mice. Stem Cells.

[11] He J, Wang Y, Lu X, Zhu B, Pei X, Wu J (2015). Micro-vesicles derived from bone marrow stem cells protect the kidney both in vivo and in vitro by microRNA-dependent repairing. Nephrology.

[12] Bruno S, Grange C, Collino F, Deregibus MC, Cantaluppi V, Biancone L (2012). Microvesicles derived from mesenchymal stem cells enhance survival in a lethal model of acute kidney injury. PLoS One.

[13] Ti D, Hao H, Tong C, Liu J, Dong L, Zheng J (2015). LPS-preconditioned mesenchymal stromal cells modify macrophage polarization for resolution of chronic inflammation via exosome-shuttled let-7b. J Transl Med.

[14] Xin H, Li Y, Liu Z, Wang X, Shang X, Cui Y (2013). MiR-133b promotes neural plasticity and functional recovery after treatment of stroke with multipotent mesenchymal stromal cells in rats via transfer of exosome-enriched extracellular particles. Stem Cells.

[15] English K, Barry FP, Mahon BP (2008). Murine mesenchymal stem cells suppress dendritic cell migration, maturation and antigen presentation. Immunol Lett.

[16] English K, Barry FP, Field-Corbett CP, Mahon BP (2007). IFN-gamma and TNF-alpha differentially regulate immunomodulation by murine mesenchymal stem cells. Immunol Lett.

[17] Jarvinen L, Badri L, Wettlaufer S, Ohtsuka T, Standiford TJ, Toews GB (2008). Lung resident mesenchymal stem cells isolated from human lung allografts inhibit T cell proliferation via a soluble mediator. J Immunol.

[18] Jackson MV, Morrison TJ, Doherty DF, McAuley DF, Matthay MA, Kissenpfennig A, et al. Mitochondrial transfer via tunneling nanotubes (TNT) is an important mechanism by which mesenchymal stem cells enhance macrophage phagocytosis in the in vitro and in vivo models of ARDS. Stem Cells. 2016. 10.1002/stem.2372 PMC4982045 27059413

[19] Li JG, Zhuan-sun YX, Wen B, Wu H, Huang FT, Ghimire H (2013). Human mesenchymal stem cells elevate CD4 + CD25 + CD127low/– regulatory T cells of asthmatic patients via heme oxygenase-1. Iran J Allergy Asthma Immunol.

[20] Mareschi K, Castiglia S, Sanavio F, Rustichelli D, Muraro M, Defedele D (2016). Immunoregulatory effects on T lymphocytes by human mesenchymal stromal cells isolated from bone marrow, amniotic fluid, and placenta. Exp Hematol.

[21] Braza F, Dirou S, Forest V, Sauzeau V, Hassoun D, Chesne J, Cheminant-Muller MA, Sagan C, Magnan A, Lemarchand P (2016). Mesenchymal stem cells induce suppressive macrophages through phagocytosis in a mouse model of asthma. Stem Cells.

[22] Gao S, Mao F, Zhang B, Zhang L, Zhang X, Wang M (2014). Mouse bone marrow-derived mesenchymal stem cells induce macrophage M2 polarization through the nuclear factor-kappaB and signal transducer and activator of transcription 3 pathways. Exp Biol Med (Maywood).

[23] Spees JL, Olson SD, Ylostalo J, Lynch PJ, Smith J, Perry A (2003). Differentiation, cell fusion, and nuclear fusion during ex vivo repair of epithelium by human adult stem cells from bone marrow stroma. Proc Natl Acad Sci U S A.

[24] Mathias LJ, Khong SM, Spyroglou L, Payne NL, Siatskas C, Thorburn AN (2013). Alveolar macrophages are critical for the inhibition of allergic asthma by mesenchymal stromal cells. J Immunol.

[25] Song X, Xie S, Lu K, Wang C (2015). Mesenchymal stem cells alleviate experimental asthma by inducing polarization of alveolar macrophages. Inflammation.

[26] Lan YW, Choo KB, Chen CM, Hung TH, Chen YB, Hsieh CH (2015). Hypoxia-preconditioned mesenchymal stem cells attenuate bleomycin-induced pulmonary fibrosis. Stem Cell Res Ther.

[27] Ni S, Wang D, Qiu X, Pang L, Song Z, Guo K (2015). Bone marrow mesenchymal stem cells protect against bleomycin-induced pulmonary fibrosis in rat by activating Nrf2 signaling. Int J Clin Exp Pathol.

[28] Tropea KA, Leder E, Aslam M, Lau AN, Raiser DM, Lee JH (2012). Bronchioalveolar stem cells increase after mesenchymal stromal cell treatment in a mouse model of bronchopulmonary dysplasia. Am J Physiol Lung Cell Mol Physiol.

[29] Lama VN, Smith L, Badri L, Flint A, Andrei AC, Murray S (2007). Evidence for tissue-resident mesenchymal stem cells in human adult lung from studies of transplanted allografts. J Clin Invest.

[30] Sinclair KA, Yerkovich ST, Chen T, McQualter JL, Hopkins PM, Wells CA, Chambers DC. Mesenchymal stromal cells are readily recoverable from lung tissue, but not the alveolar space, in healthy humans. Stem Cells. 2016. 10.1002/stem.2419 27352824

[31] Sabatini F, Petecchia L, Tavian M, Jodon de Villeroche V, Rossi GA, Brouty-Boye D (2005). Human bronchial fibroblasts exhibit a mesenchymal stem cell phenotype and multilineage differentiating potentialities. Lab Invest.

[32] Dominici M, Le Blanc K, Mueller I, Slaper-Cortenbach I, Marini F, Krause D (2006). Minimal criteria for defining multipotent mesenchymal stromal cells. The International Society for Cellular Therapy position statement. Cytotherapy.

[33] Rohart F, Mason EA, Matigian N, Mosbergen R, Korn O, Chen T, Butcher S, Patel J, Atkinson K, Khosrotehrani K (2016). A molecular classification of human mesenchymal stromal cells. PeerJ..

[34] Goddette DW, Frieden C (1986). Actin polymerization. The mechanism of action of cytochalasin D. J Biol Chem.

[35] Badri L, Walker NM, Ohtsuka T, Wang Z, Delmar M, Flint A (2011). Epithelial interactions and local engraftment of lung-resident mesenchymal stem cells. Am J Respir Cell Mol Biol.

[36] Sagar GD, Larson DM (2006). Carbenoxolone inhibits junctional transfer and upregulates Connexin43 expression by a protein kinase A-dependent pathway. J Cell Biochem.

[37] Desplantez T, Verma V, Leybaert L, Evans WH, Weingart R (2012). Gap26, a connexin mimetic peptide, inhibits currents carried by connexin43 hemichannels and gap junction channels. Pharmacol Res.

[38] Evans WL, Leybaert L (1997). Mimetic peptides as blockers of connexin channel-facilitated intercullar communication. Cell Commun Adhes.

[39] Chambers DC (2015). In the end it’s a replication problem: what measuring telomere length can tell us about idiopathic pulmonary fibrosis. Respirology.

[40] Wilson JG, Liu KD, Zhuo H, Caballero L, McMillan M, Fang X (2015). Mesenchymal stem (stromal) cells for treatment of ARDS: a phase 1 clinical trial. Lancet Respir Med.

[41] Karoubi G, Cortes-Dericks L, Breyer I, Schmid RA, Dutly AE (2009). Identification of mesenchymal stromal cells in human lung parenchyma capable of differentiating into aquaporin 5-expressing cells. Lab Invest.

[42] Wagner W, Wein F, Seckinger A, Frankhauser M, Wirkner U, Krause U (2005). Comparative characteristics of mesenchymal stem cells from human bone marrow, adipose tissue, and umbilical cord blood. Exp Hematol.

[43] Pelekanos RA, Li J, Gongora M, Chandrakanthan V, Scown J, Suhaimi N (2012). Comprehensive transcriptome and immunophenotype analysis of renal and cardiac MSC-like populations supports strong congruence with bone marrow MSC despite maintenance of distinct identities. Stem Cell Res.

[44] Badri L, Murray S, Liu LX, Walker NM, Flint A, Wadhwa A (2011). Mesenchymal stromal cells in bronchoalveolar lavage as predictors of bronchiolitis obliterans syndrome. Am J Respir Crit Care Med.

[45] Walker N, Badri L, Wettlaufer S, Flint A, Sajjan U, Krebsbach PH (2011). Resident tissue-specific mesenchymal progenitor cells contribute to fibrogenesis in human lung allografts. Am J Pathol.

[46] Guan XJ, Song L, Han FF, Cui ZL, Chen X, Guo XJ (2013). Mesenchymal stem cells protect cigarette smoke-damaged lung and pulmonary function partly via VEGF-VEGF receptors. J Cell Biochem.

[47] Ko JH, Lee HJ, Jeong HJ, Kim MK, Wee WR, Yoon SO (2016). Mesenchymal stem/stromal cells precondition lung monocytes/macrophages to produce tolerance against allo- and autoimmunity in the eye. Proc Natl Acad Sci U S A.

[48] Crespin S, Bacchetta M, Bou Saab J, Tantilipikorn P, Bellec J, Dudez T (2014). Cx26 regulates proliferation of repairing basal airway epithelial cells. Int J Biochem Cell Biol.

[49] Hong KU, Reynolds SD, Watkins S, Fuchs E, Stripp BR (2004). Basal cells are a multipotent progenitor capable of renewing the bronchial epithelium. Am J Pathol.

