# Supplementary figures and images for: Characterization of intercellular communication and mitochondrial donation by mesenchymal stromal cells derived from the human lung
Source: Stem Cell Res Ther. 2016 Jul 12;7:91. doi: 10.1186/s13287-016-0354-8 (PMC4942965; doi:10.1186/s13287-016-0354-8)

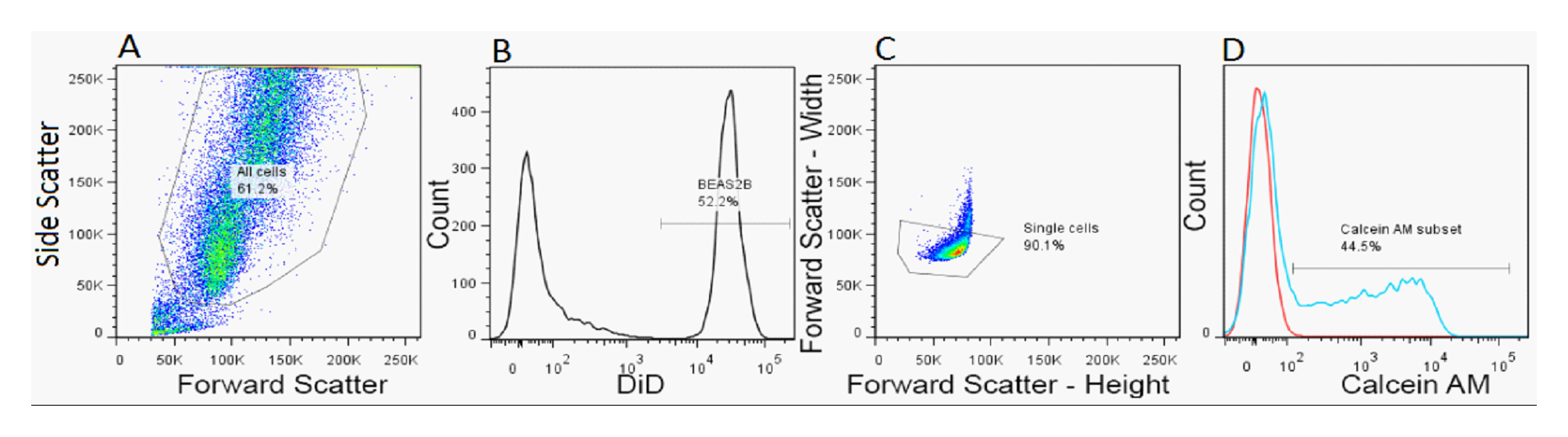

Supplement: Additional file 1: Figure S1. — Showing the gating strategy for measuring BEAS2B Calcein AM fluorescence after co-culture. MFI of Calcein AM within BEAS2B cells was determined by flow cytometry. (a) Forward/side scatter gating was used to exclude debris. (b) BEAS2B epithelial cells were then selected based on DiD fluorescence. (c) Doublets were then eliminated using forward scatter—width and height. (d) A fluorescence minus one control (red line) was used to distinguish positive Calcein AM and negative BEAS2B cells. (TIF 481 kb) [file 13287_2016_354_MOESM1_ESM.tif]

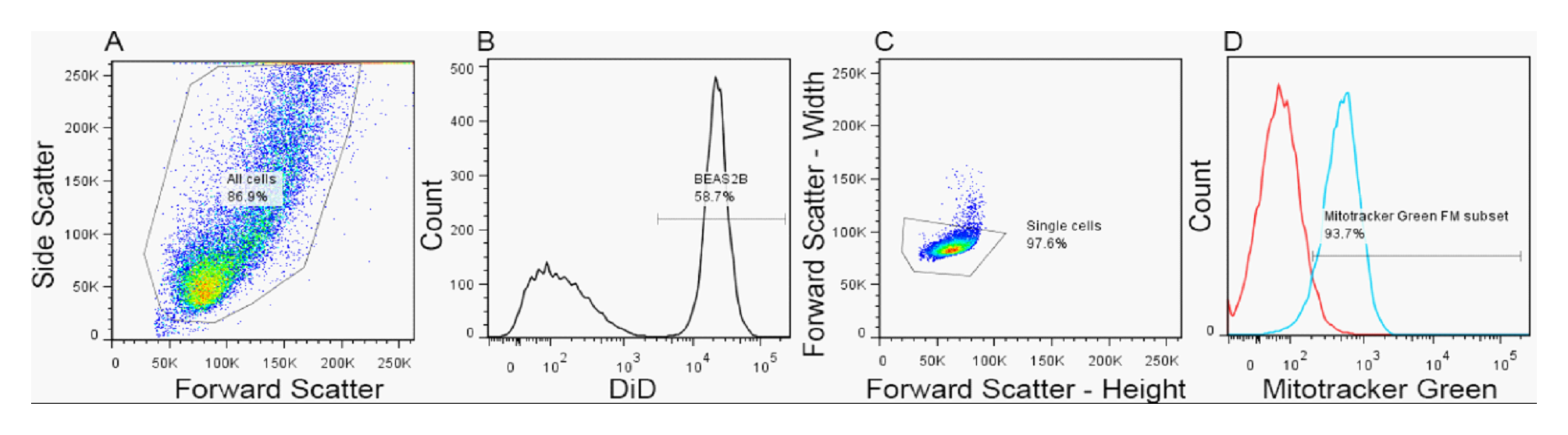

Supplement: Additional file 2: Figure S2. — Showing the gating strategy for measuring BEAS2B Mitotracker Green fluorescence. MFI of Mitotracker Green within BEAS2B cells was determined by flow cytometry. (a) Forward/side scatter gating was used to exclude debris. (b) BEAS2B epithelial cells were then selected based on DiD fluorescence. (c) Doublets were then eliminated using forward scatter—width and height. (d) A fluorescence minus one control (red line) was used to distinguish positive Mitotracker Green and negative BEAS2B cells. (TIF 461 kb) [file 13287_2016_354_MOESM2_ESM.tif]

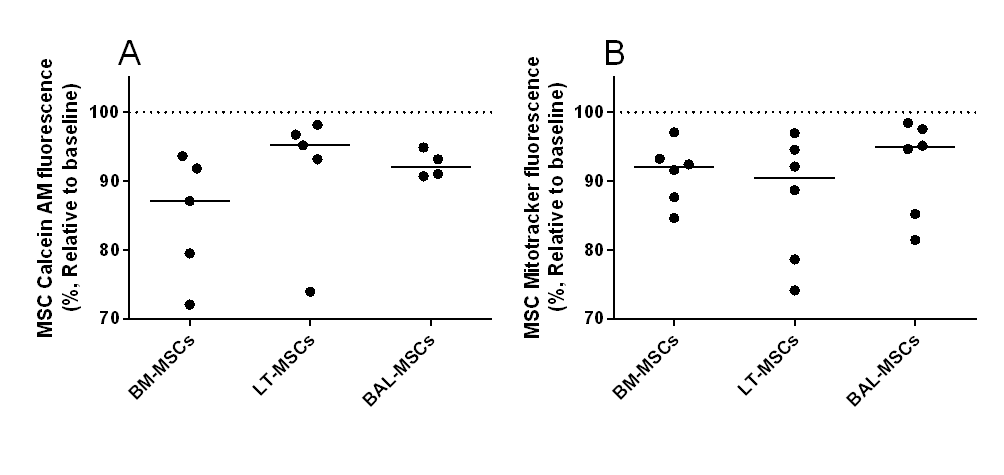

Supplement: Additional file 4: Figure S3. — Showing that MSC Mitotracker Green fluorescence decreases after co-culture with BEAS2B epithelial cells. The change in mesenchymal stromal cell fluorescence (a Calcein AM, b Mitotracker Green) after co-culture with BEAS2B epithelial cells. Dotted horizontal line represents baseline calculated as the MFI of MSC grown in homogenous cultures. Solid horizontal line represents median. (TIF 20 kb) [file 13287_2016_354_MOESM4_ESM.tif]
