# Supplementary material for: Characterization of intercellular communication and mitochondrial donation by mesenchymal stromal cells derived from the human lung
Source: Stem Cell Res Ther. 2016 Jul 12;7:91. doi: 10.1186/s13287-016-0354-8 (PMC4942965; doi:10.1186/s13287-016-0354-8)
Supplement: Additional file 3: Table S1. — Presenting supplementary methods. (DOCX 12 kb) [file 13287_2016_354_MOESM3_ESM.docx]

**Supplementary Methods, Table 1**

| Name | Manufacturer | Target | Working concentration |
| --- | --- | --- | --- |
| Cytochalasin D | Sigma-Aldrich | Micro/nanotubules | 350nM |
| Dynasore | BioVision | Endocytosis | 80µM |
| GAP26 | Apex Biotechnology | Connexin-43 | 160-320µM |
| Carbenoxelone | Sigma-Aldrich | All GAP junctions | 100µM |
| Vehicle control – DMSO | Sigma-Aldrich |  | 1µL/mL (volume matched) |
